# Editorial: Encoding and Navigating Linguistic Representations in Memory

**DOI:** 10.3389/fpsyg.2017.00164

**Published:** 2017-02-09

**Authors:** Claudia Felser, Colin Phillips, Matthew Wagers

**Affiliations:** ^1^Potsdam Research Institute for Multilingualism, University of PotsdamPotsdam, Germany; ^2^Department of Linguistics, University of MarylandCollege Park, MD, USA; ^3^Language Science Center, University of MarylandCollege Park, MD, USA; ^4^Department of Linguistics, University of California, Santa CruzSanta Cruz, CA, USA

**Keywords:** sentence comprehension, encoding, memory retrieval, interference, anaphor resolution, agreement processing, filler-gap dependencies

## Motivations

We created this research topic to address two closely related needs: to support a rapidly growing area of language science, and to support the (predominantly young) scientists who are working in this area. Recent years have seen a rapid growth in the amount of psycholinguistic research being carried out in linguistics departments. This has created a venue for exploring new questions. Understanding structured mental representations and the relations within them is the bread-and-butter of much research in linguistics, but the traditional focus has been on theories at a level of analysis that assumes discrete, symbolic representations, and is agnostic about how those representations are constructed in real time, whether in comprehension, production, or acceptability judgment tasks. Now there is a community of researchers who are working to understand these phenomena in more fine-grained terms.

In the area of syntax, the growth in research at the intersection of linguistics and psychology has been fueled by a number of parallel developments.

First, by connecting linguistic representations with psychological theories of memory encoding and access. The literature on memory encoding provides only limited inspiration for theories of structured linguistic representations, because most memory research is based on unstructured lists. But the literature on memory access has served as a strong inspiration for theories of linguistic dependency formation. In particular, models of content-addressable memory (CAM) have been influential in psycholinguistics. In CAM, items in memory are accessed (or their activation-level is boosted) based on their match to a set of content-based retrieval cues, rather than based on their memory address, as in classical computational architectures. A hallmark of memory access in CAM is similarity-based interference effects. These effects have been widely documented in language processing (Gordon et al., 2001; Van Dyke, 2007) and they feature prominently in many of the papers in the current collection. A second hallmark of memory access in CAM is non-effects of structural or linear distance in retrieval times (McElree et al., 2003), and these effects are the focus of one article in this collection (Dillon et al.). The influence of CAM on psycholinguistics has been aided by an implemented CAM-based parsing model (ACT-R: Lewis and Vasishth, 2005). This model makes specific, testable predictions, and provides a useful framework for thinking about memory access in language processing.

Second, research on the time course of linguistic processes is now more accessible, due to portable and affordable technical resources. Many studies can be carried out on laptop computers or even via the internet. Free statistical software packages are widely used, aided by a supportive user community. And one should not underestimate the value of role models that show that linguists can do this kind of research.

Together, the theoretical and practical developments have opened up a playground of languages and linguistic phenomena that can be used to develop and test models of real-time linguistic processes. The scale and diversity of the research in this collection was not possible 10–15 years ago.

However, publication venues have not kept pace with the growth in research at the intersection of linguistics and psychology. Researchers in this area still need to choose whether to submit their work to journals with a traditional linguistics focus or journals with a traditional psychology focus. For example, the *Journal of Memory and Language* is one of the most highly regarded journals in psycholinguistics. As of the start of 2017 its editorial staff and editorial board include more than 50 individuals, and there is almost no representation from linguistics. The associate editorial board of *Linguistic Inquiry*, an influential linguistics journal, has 70 members and just a couple of psycholinguists (To its credit, the new open access linguistics journal *Glossa* has a more diverse editorial team). This polarization means that there are few hospitable outlets for research that is unapologetic in its use of both linguistic and psychological models and analyses. Psychology journals routinely tell authors that their work “will not be understandable to our readers,” even for relatively basic linguistic notions, especially if not from English. Notice the quaint idea that people read journals rather than articles. Linguistics journals are more likely to ask authors, “But how does this bear on theory?,” where “theory” is assumed to mean claims at the traditional level of linguistic analysis, as if psycholinguists don't build theories. Of course, a number of papers have made their way into prominent journals in either field (e.g., Sturt, 2003; Phillips, 2006), but there is limited appetite for ongoing debates that delve into the details of real-time grammatical processes.

An additional benefit of editing this research topic is that we have been pleasantly surprised by the effectiveness of the Frontiers editorial process, which departs from tradition in a number of respects.

- As research topic editors, we could request 1-page abstracts before inviting a full paper submission. This allowed efficient triage of unsuitable submissions, and allowed us to make suggestions to authors at an early stage in the process.
- The focus in the review process on soundness rather than impact was liberating for authors and reviewers alike, as they could engage more candidly with one another about what the results did and did not show. There has been much recent hand-wringing about transparency and replicability in psychological science (Open Science Collaboration, 2015). Most discussions have focused on how to avoid problems by legislating pre-registration, data sharing, specific analyses, etc. But it's just as important to remove the unrealistic expectations that create pressure on authors to distort their claims (Nosek et al., 2012; Maner, 2014). Our experience with this research topic is that authors are refreshingly open when the system does not penalize them for that.
- The interactive review process helped to keep reviewers and authors on target and on time. The discussion-like format also made the process more collegial.

The success of the research topic is also evident in various metrics. As of January 2017, the 48 articles in this collection are the largest Frontiers research topic under the heading Psychology (out of 482 research topics) and Language Sciences (out of 53 research topics). One hundred and fifty-five authors contributed, as did 74 reviewers. This leaves little doubt that there is a market for publication venues at the intersection of linguistics and psychology. Also, the publication cycles were dramatically faster than other journals in linguistics and psycholinguistics. The median time from manuscript submission through two rounds of review (independent + interactive) to online publication was just over 4 months (128 days). For traditional journals the norm is 1–2 years or more. Of course, rapid publication is particularly valuable for junior researchers whose careers are hurt by extremely slow publication cycles. The speed of publication had the unexpected consequence that articles in this collection were cited at rates that are competitive with leading journals in linguistics, although this was not part of our original goal. We refer interested readers to a fuller discussion of our editorial experience elsewhere (Phillips, 2016).

Although we were encouraged by the number and quality of the articles and by the success of the review process, there were a couple of areas where we were disappointed. We hoped to see more articles on populations other than young adult native speakers, and we hoped to see more computational contributions. In both of these areas the lower demand may reflect the availability of other accessible and fast outlets. We also hoped to see more articles focusing on theory and synthesis. If this new field of research is to be sustainable, then it will need more than a large body of findings about specific linguistic phenomena in diverse languages. Without theoretical debates that serve to organize and guide research, the field will quickly run out of steam.

## Topical review

To review the individual contributions in our research topic, we have divided our collection roughly by linguistic phenomena, and consider the (overlapping) subsets of articles that deal with *anaphor resolution, filler-gap dependencies*, and *agreement*. These subsets, particularly the first two, address questions of how structured, compositional information is used in online sentence comprehension, and what kinds of features or similarity relations can aid or hinder comprehension. These themes—particularly the latter two—also consider how forward-looking the parser is, both in terms of expectations it explicitly commits to, but also in terms of how it encodes present information for future retrieval.

## Anaphor resolution

Nineteen of the articles in the collection focus on anaphor resolution (And that does not include the articles on different forms of ellipsis). Encountering an anaphoric expression is thought to trigger a memory search for a suitable antecedent. Of the linguistic phenomena represented in this collection, anaphor resolution seems the most obviously suitable one for testing theoretical assumptions about memory access and retrieval, and how different types of cue interact in guiding the search process. Linguistic constraints on anaphor resolution such as Conditions A and B of the binding theory (Chomsky, 1981; Sportiche, 2013) seem to be at odds with some of the assumptions of well-motivated retrieval models. Most of the studies on anaphor resolution in this collection focus on interference; that is, on the question of whether anaphor resolution is affected by the presence of feature-matching distractors, and whether this is the case even for distractors that are structurally illicit antecedents. Interference can serve as a probe for memory access mechanisms. Whilst earlier studies investigating the role and timing of binding constraints during anaphor resolution mostly focused on English or English-type reflexives and pronouns (e.g., Nicol and Swinney, 1989; Badecker and Straub, 2002; Sturt, 2003), the articles in the current collection considerably expand the empirical research base by examining other languages and types of anaphora, including bound variable and long-distance anaphora.

A number of articles investigate the processing of reflexives or reciprocals. Using the visual-world paradigm, Clackson and Heyer find evidence for similarity-based interference during the processing of English reflexives, with listeners being distracted by a discourse-prominent but syntactically inaccessible antecedent. Patil et al. observe interference effects during the processing of English reflexives using eye-movement monitoring during reading, and Jäger et al. report interference effects during the processing of reflexives in Mandarin. No interference effects were observed by Kush and Phillips in the processing of pre-verbal anaphors (reciprocals) in Hindi, however. Jäger et al. report a series of studies on German and Swedish reflexives, where their findings suggest that interference affects retrieval but not encoding.

Dillon et al. provide experimental and modeling evidence for a locality bias for Mandarin long-distance reflexives, using a speed-accuracy tradeoff (SAT) paradigm, and Dillon et al. show this bias to be reduced for morphologically complex reflexives. Also examining Mandarin reflexives, He and Kaiser provide reading-time evidence showing that person features can block long-distance referential dependencies. Frazier et al.'s eye-movement results show that syntactic gaps (*wh*-traces) interact with reflexive resolution in English.

Several other contributions focus on non-reflexive pronouns. Looking at pronoun resolution across sentence boundaries, Autry and Levine demonstrate that multiple distractors give rise to cumulative (“fan”) effects. Schumacher et al. examine the inter-sentential resolution of German pronouns and demonstratives using ERPs. Their results suggest that both semantic and positional cues contribute to a potential antecedent's referential prominence.

Investigating the role of binding constraints during pronoun resolution, Chow et al. present reading-time evidence which indicates that the antecedent search is constrained by Condition B. This conclusion is further supported by the findings reported by Patterson et al., whose participants also included non-native speakers of English. Unlike the native group, the non-native speakers showed a bias toward matrix subject antecedents, regardless of whether or not local coreference was allowed. The eye-movement results reported by Cunnings et al. show that c-command constrains pronoun binding by a quantificational antecedent, but not coreference between a pronoun and a non-quantificational antecedent. These findings are in line with what Sportiche (2013: 196) has dubbed “Condition D” of the binding theory. Pablos et al. investigate the processing of Dutch cataphoric (rather than anaphoric) pronouns using ERPs. Their results indicate that binding Condition C constrains the search for a suitable referent. The contribution by Parker et al. provides evidence for similarity-based interference during the computation of adjunct control dependencies, showing that an overt pronoun is not necessary.

Finally Koornneef and Reuland's “hypothesis and theory” article draws largely on findings from anaphor resolution studies. The authors argue that “deep” or grammatically driven processing is not necessarily computationally more costly than “shallow” processing using extra-grammatical information sources.

The studies on anaphora in this collection ultimately present a mixed picture: On the one hand, there is clear evidence of structure-based constraints guiding the antecedent search, while on the other hand referential dependency formation has been shown to be vulnerable to similarity-based interference under specific conditions. These seemingly contradictory findings can possibly be accounted within CAM-based processing models capable of implementing structure-sensitive constraints, and the specific way of capturing them is a focus of current debate.

## Filler-gap dependencies

A large number of articles in this collection examine the processing of filler-gap dependencies, with most of them focusing on *wh*-movement or relative clauses. Filler-gap dependencies are mediated by hierarchical phrase structure representations, and successfully completing them involves both memory storage and retrieval (e.g., Gibson, 1998). Encountering a filler such as *which student* in a *wh*-interrogative sentence like *Which student did you say you met at the concert last night?* is thought to trigger the prediction of a corresponding gap to which the filler must be linked before it can be fully integrated into the emerging sentence representation. Piñango et al. present brain imaging evidence showing that gap search and gap completion processes can be distinguished at the neurocognitive level.

As a filler needs to be kept in memory until a suitable gap can be identified, processing filler-gap dependencies can incur measurable storage costs. The difficulty of retrieving a filler (or antecedent) at a gap site may be affected by the nature of the sentence material that intervenes between antecedent and gap, giving rise to interference effects. Stepanov and Stateva report cross-linguistic reading-time evidence for memory storage effects during the processing of *wh*-adjunct dependencies in both English and Slovenian, and Santi et al. present brain-imaging results which show that *wh*-dependency formation is affected by the syntactic type of the intervening sentence material. Using the visual-world paradigm, Haendler et al. show that German-speaking children's ability to comprehend object relative clauses is affected by referential properties of the intervening subject. In their Methods article, Sekerina et al. demonstrate that items held in short-term memory can interfere with filler retrieval during the auditory processing of object clefts, replicating earlier findings from reading-based tasks in a different modality. Using eye-movement monitoring during reading, Sturt and Kwon show that the processing of both subject raising and nominal control dependencies is subject to facilitatory interference effects. Also taking into account Parker et al.'s findings of interference effects during the computation of adjunct control dependencies, these studies indicate that antecedent retrieval at gap sites is generally vulnerable to interference.

This conclusion is further corroborated by the two studies in this collection that have investigated sluicing, a special type of clausal ellipsis that involves fronting of a remnant *wh*-expression (as in *He lost his keys but didn't know where*). The eye-movement data presented by Harris provide evidence that antecedent retrieval during the processing of sluiced sentences is subject to similarity-based interference modulated by structural properties of the antecedent. The contribution by Paape examines how the presence of a temporary subject/object ambiguity in the antecedent affects the processing of sluiced sentences in German.

Two further studies have examined effects of individual differences in working memory (WM) capacity on the processing of filler-gap dependencies. Nicenboim et al. present reading-time evidence for such effects from Spanish, and Nicenboim et al. report further evidence showing that locality effects in Spanish and German are modulated by WM capacity.

Several contributions focus on the nature of the gap search and how this search is constrained, or on the question of how dependency formation interacts with other grammatical computations. Omaki et al.'s findings show that the gap search process is highly predictive, with direct object gaps being postulated independently of verb transitivity even in a verb-medial language like English. The contribution by Lin focuses on the role of expectation during the processing of different types of subject relative clauses in Chinese, showing that canonical thematic ordering facilitates processing. Leiken et al. investigate the role of gap predictability in the processing of English object relatives (in comparison to verb-phrase ellipsis and right-node raising structures) using magnetoencephalography. Their results suggest that the left-anterior frontal gyrus (LIFG), a brain region previously found to be involved in dependency formation, subserves memory retrieval at gap sites regardless of whether or not the gap was predictable. Franck et al. use the phenomenon of agreement attraction to demonstrate that computing filler-gap dependencies involves the creation of abstract hierarchical phrase-structure representations, and Frazier et al. demonstrate that *wh*-gaps interact with the processing of reflexives. Engaging in an active gap search does not mean that gaps are postulated freely, however. The reading-time results reported by Johnson et al. suggest that neither native English speakers nor native Korean-speaking learners of English postulate gaps in so-called “island” environments.

Other studies examine how properties of the filler affect the processing of filler-gap dependencies. Atkinson et al. report a series of acceptability judgment experiments showing that morphosyntactic and semantic features interact in ameliorating *wh*-island violations, and Goodall shows that one of the factors known to ameliorate island violations (“d-linking”) also improves the acceptability of non-island sentences. Hofmeister and Vasishth's reading-time results indicate that more complex fillers are easier to retrieve at gap sites than less complex ones, and the findings reported by Troyer et al. show that elaboration also facilitates filler retrieval across short pieces of discourse.

Taken together, the above studies provide strong evidence that retrieval at gap sites is vulnerable to similarity-based interference, that more complex or elaborate fillers are easier to retrieve than less complex ones, and that both gap postulation and filler retrieval are sensitive to information encoded in hierarchical phrase-structure representations.

## Computing agreement and feature-based encoding

Several articles in our collection address the phenomenon of agreement attraction. Agreement attraction is a robust perceptual illusion that mirrors a speech error in production (Bock and Miller, 1991). For example, speakers are prone to produce sentences like *The **dogs** [that the shelter**rescue** in the winter] eventually got adopted*. In our example, the agreement on the verb *rescue* should be controlled by its singular, grammatical subject, *the shelter* but instead it is attracted to agree with another nearby phrase *the dogs*—the attractor. Not only are such examples easily found in natural speech and elicited in the lab, but they are also routinely missed by language perceivers: speakers experience an illusion of grammaticality.

The illusion turns out to be very sensitive to the relationship between the features on the grammatical subject, the attractor noun, and the verb. For this reason, it has served as a productive system for probing issues around linguistic encoding: what are the features with which comprehenders represent partial linguistic information in their working memory? A common, well-motivated view about how nouns are encoded relies on feature markedness. Along any feature scale, certain values are more marked than others, like plural is more marked than singular: for example, plural nouns are less common than singular nouns and are usually signaled by more complex morphological forms (*dog-s* vs. *dog*-Ø). And it appears that these marked values are more visible in the comprehension system, in terms of how they are encoded during language processing (Eberhard, 1997). But several papers in our collection demonstrate that a more nuanced view is necessary, and they do so by looking at feature systems in under-investigated languages with grammars that have more complicated syntax/morphology mappings, ones that are more amenable to investigating this question.

Tucker et al. show that in Modern Standard Arabic (MSA) the morphological exponence of a marked feature also matters. MSA expresses the plural feature in two ways, the so-called suffixed plural and the broken (or ablaut) plural. The suffixed plural causes more attraction and with a different time-course than the ablaut plurals in MSA. Slioussar and Malko examined a more complex feature system—gender—in Russian. The Russian gender system has three values, called masculine, feminine, and neuter. This three-way distinction makes it possible to demonstrate that, in determining whether attraction will occur, the visibility of the attractor alone is insufficient, and the visibility of the head is crucial. Nicol et al. also reinforce the conclusion that the head-attractor relationship matters, but from the perspective of hierarchical relatedness. Attractors that are closer to the head noun in the sentence's phrase structure induced more attraction effects. Moreover, how saliently a noun was marked as non-nominative mattered (e.g., *women's* generated less attraction than *dogs'*). Franck et al.'s paper on filler-gap dependencies likewise demonstrates that the relative syntactic prominence of the attractor is encoded.

Research on how plurals are encoded in real-time not only feeds-back into how plurality is represented in the grammar, but it also leads the way to broader questions of the relationship between *how* dependent elements are initially encoded and how retrieval cues are identified and integrated across time. These issues are taken up by Tucker et al. in their discussion of “feature-cue” algorithms in MSA. But Martin's Hypothesis article raises higher-level questions about how a theory of cue integration might relate different kinds of linguistic representation, such as how information signaled by distinct morphemes may be integrated into the percept of a phrase. Riordan et al. specifically consider the cue validity of Number in a broad variety of syntactic contexts; they demonstrate that number cues often generate quite weak predictions about numerosity, based on anticipatory looking in the visual world paradigm.

Hofmeister and Vasishth address the encoding/retrieval relation from a different angle: investigating the processing of relative clauses, they show that only syntactic and semantic elaboration of an left dependent (the RC head) affects retrieval at the right dependent (the RC verb)—but other differences experienced at encoding, like different text colors, do not. Troyer et al. show that such elaborative effects can happen as referents are processed over the span of a discourse, and not merely locally, i.e., not only when the elaboration occurs at the targeted retrieval position itself.

When there are evidently effects of similarity-based interference at a retrieval site, it is important to assess whether such effects derive from the process of retrieval itself or whether they might have arisen during the process of encoding. Along these lines, Häussler and Bader argue that interference at retrieval can explain the “missing VP” effect observed in the processing of center self-embedded relative clauses, even in languages like German which may benefit from highly predictive encoding mechanisms. Likewise, Jäger et al.'s paper on reflexives also takes up a phenomenon to argue that the culprit in any online fallibility is explicitly not encoding interference.

The remaining articles in this collection address issues relating to prediction, memory retrieval, or the role of linguistic structure in dependency formation by examining other linguistic phenomena. Brusini et al.'s contribution investigates verb prediction in French, providing evidence that syntactic cues constrain lexical access. In a series of reading-time experiments, Safavi et al. examine the processing of complex predicates in Persian, showing that dependency resolution difficulty is affected both by predictability and distance. Using an auditory speed-accuracy tradeoff paradigm, Johns et al. show that individual differences in reading skill do not affect memory retrieval during listening. McCourt et al. present reading-time evidence which challenges previous claims to the effect that the phenomenon of implicit control involves a silent syntactic argument. Xiang et al. show that susceptibility to interference during the processing of negative polarity items (NPIs) correlates with pragmatic reasoning as measured by an autism scale, whereas susceptibility to agreement attraction does not correlate. This indicates that NPI illusions have a different source than agreement attraction.

## Author contributions

All authors listed have made substantial, direct and intellectual contribution to the work, and approved it for publication.

## Funding

Our work on this Editorial has been supported by an Alexander-von-Humboldt Professorship to Harald Clahsen, by NSF grant DGE-1449815 to CP, and by NSF grant BCS-1251429 to MW.

### Conflict of interest statement

The authors declare that the research was conducted in the absence of any commercial or financial relationships that could be construed as a potential conflict of interest.
